# Staying Connected: Reaching Out to Psychiatric Patients During the Covid-19 Lockdown Using an Online Blog

**DOI:** 10.3389/fpubh.2020.592618

**Published:** 2020-12-17

**Authors:** Astrid Lehner, Klaus Nuißl, Winfried Schlee, Berthold Langguth

**Affiliations:** Department for Psychiatry and Psychotherapy, University of Regensburg, Regensburg, Germany

**Keywords:** SARS-CoV-2, COVID-19, corona virus, clinical psychiatry, pandemic, e-health, citizen involvement, experienced involvement

## Abstract

Health systems worldwide are challenged by the coronavirus pandemic and all medical specialties have struggle to meet the conflicting requirements for virus containment on the one hand and treatment of other medical conditions on the other. This holds true also for psychiatry. *Per se*, psychiatric patients are highly vulnerable to suffer from social isolation and loneliness. As a result of the Covid-19 pandemic and lockdown measures, unfortunately, this vulnerability is even further increased. As a part of its pandemic risk management, the outpatient clinic of the Psychiatric District Hospital Regensburg launched an online blog as a means of assisting patients who were required to stay home. Aim of the blog was to stay by patients' side in those uncertain times by offering an online connection to their therapists, by providing important information about the pandemic situation, by offering some ideas on how to build a daily routine and how to meaningfully spend their time at home during the lockdown. We also aimed at involving patients as experts in their own affairs by inviting them to contribute to the blog's shape and content. As a result of coordinated team effort, it was possible to launch a blog within few days, and this was perceived helpful by many patients. Overall, however, patient involvement turned out to be a challenge requiring more attention in future work.

## Introduction

In order to slow down the rapid spread of SARS-CoV-2, many countries worldwide have to face restrictions in public and private life. For health care professionals and hospitals, this implies the challenge of balancing preventive measures to stop the spread of the virus and, at the same time, address the needs of patients suffering from other medical conditions.

Patients with serious psychiatric illness are known to be particularly vulnerable during crises of health care systems (1, 2). Recently, it has been shown that patients with mental disorders do have an increased risk of Covid-19 infection and mortality (3). There have already been some suggestions on how to manage in- and outpatient units during the pandemic in order to provide the best possible treatment (2, 4). For outpatient treatment, this includes the limitation of face-to-face appointments and the shift toward telemedicine wherever possible (5, 6).

As psychiatry is mostly based on verbal, paraverbal and nonverbal communication and less on analytical tests or physical examination, video or telephone appointments are easier to accomplish in psychiatry compared to many other medical professions (7). Furthermore, the repertoire of telemedicine is expanded by a growing amount of online programs and apps offering support for patients suffering from mental illness (8). In the last decade, telemedicine has been increasingly examined in outpatient care of psychiatric patients and has been shown to have comparable outcomes as in-person treatment e.g., for patients suffering from depression (9). Telemedicine has also been shown to improve clinical outcomes for patients suffering from post-traumatic stress disorder by supporting them to initiate and ideally complete evidence-based psychotherapy (10). Furthermore, telemedicine enables continuous psychiatric care for patients from more rural areas who might otherwise not be able to attend appointments on a regular basis (11).

With respect to the Covid-19 pandemic, telepsychiatry has been reported to be well-accepted by patients during phases of public lockdown (12). It involves phone calls and video-based appointments both of which are able to compensate for one-to-one contacts between patients and health care professionals.

However, pandemic risk management often includes the complete shutdown of group therapies (4), an important part of psychiatric treatment. It is much more complicated to cover those group activities by phone or video calls due to technical challenges and digital privacy protection issues. Consequently, in many cases, those therapies are paused without replacement during phases of lockdown. This applies e.g., to occupational therapy, sports therapy or musical therapies, often an integral part of patients' daily routines, especially when suffering from severe or chronic psychiatric conditions. Many of those patients live socially isolated and have difficulties keeping a stable daily routine in general, and during public lockdown in particular, increasing the risk of symptom deterioration.

Corona risk management in the Psychiatric District Hospital Regensburg, Germany, also included the reduction of such group activities for inpatients and the complete cancellation of group therapies for outpatients (13). As patients reported facing problems staying at home and feeling deserted by their health care professionals, therapists from the outpatient clinic joined forces with patient representatives and the hospital's Public Relations department to launch a patient-centered online Blog as an add-on to phone-calls and video-meetings. Aim of the blog was to make patients feel connected to the clinic and their therapists, to provide them with some ideas on how to build a daily routine and how to meaningfully spend their time at home in those uncertain times. We also tried involving patients as experts in their own affairs, with the objective to allow them to contribute to and shape the blog's content. A colleague with a degree in “experienced involvement,” a German training for peer support, was involved in implementing the blog. Peer support has been shown to improve self-efficacy, sense of hope and quality of life of patients with mental disorders (14, 15).

## Methods

Through this paper, we provide information about the launching and content of the blog and about adjustments we had to make. In addition, the total number of page views will be reported. Only page views from outside the clinic are counted, i.e., page views inside the clinic's network were'nt taken into consideration. Furthermore, mean page views on working days (Monday to Friday) were compared with mean page views on the weekend using an unpaired *t*-test. IBM SPSS Statistics 24 (IBM Corporation, Armonk, NY) was used for data analyses.

The main target group of the blog was a subgroup of the clinic's outpatients. The outpatient department serves over 8,000 patients/year. However, the blog targeted primarily patients, who suffer from chronic psychiatric disorders such as affective disorders, schizophrenia or personality disorders, and who receive regular multidisciplinary treatment in our outpatient clinic. This group comprises about 300 patients. As the information about the establishment of the blog were communicated through many different channels, it is very difficult to estimate, how well we reached the target group and how many more patients received the information. As the blog was open to the public and could therefore be used and read by anyone interested, the users of the blog are hereinafter referred to as “readers” instead of “patients.”

Furthermore, readers' feedback will be reported including feedback given during phone calls or by email, as well as feedback provided during an online survey. Readers were continuously encouraged to write emails in case of questions or ideas concerning the blog. After 7 weeks of blog activity, we asked for readers' wellbeing and their feedback in an anonymous online survey that was approved by the Ethics Committee of the University Hospital of Regensburg, Germany (reference number 20-1862-101). In order to facilitate the survey, there was an explanatory blog post, also providing the link to the questionnaire. Thereby, we ensured that only blog readers could participate in the survey. The survey was generated and launched using the services of www.soscisurvey.de.

In this survey, we asked for readers' age, gender, place of residence, current or past experience of a psychiatric illness and whether the reader had been under medical treatment in the Psychiatric District Hospital Regensburg. Furthermore, a modified version of the Clinical Global Impression (16) was used to enquire about any changes in subjective clinical global impression of somatic and mental wellbeing in comparison to before the beginning of lockdown measures (from 1 = much better to 7 = much worse) and for unexpected improvements or impairment in everyday life due to public lockdown. With respect to the blog, we asked (a) for how many weeks the blog had already been known by the reader, (b) for how many weeks the blog was actively read, (c) how useful the blog was perceived to be, (d) how often the blog was visited, (e) how often the new articles were read and, (f) how often information and tips were actively transferred into everyday life. Finally, we asked which aspects of the blog were perceived as being useful. The readers' feedback will be presented descriptively.

## Results

### Blog Content

The blog comprised two categories: The first category contained general information about the Covid-19 pandemic and rules of conduct, as well as tips for everyday life. For instance, there was a video on face masks, links to reliable sources of information regarding the coronavirus pandemic and a blog post on how to explain the current situation to children. These articles could be read anytime. The second category consisted of two articles per day providing tips given by different professions: psychotherapeutic tips, mindfulness, recovery, occupational therapy, healthy and easy recipes, ideas for home workouts, musical therapy and positive news. Furthermore, we tried to foster peer support by asking patients what was helpful for them during their daily life in this period of lockdown. The incoming information was then shared anonymously on the blog.

The articles consisted of written information, links to online resources and amateur audios and videos created by the clinic's health care staff. As many of the regular treatment offers paused, it was possible for the staff to invest a certain amount of time for producing blog content. Thereby, it was ensured that patients saw familiar faces and heard familiar voices in those uncertain times, whilst staying at home. Additionally, readers could write an email if they had questions with respect to the content of the articles.

There were two new articles per day from Monday to Friday, published on 9:00 a.m. and 3:00 p.m. These articles could only be retrieved for 1 h (i.e., until 10:00 a.m. and 4:00 p.m., respectively) in order to provide a certain daily routine. On Saturdays, there was one article at 3:00 p.m. On Sundays and bank holidays, there were no regular articles published.

### Timeline and Adjustments

On March 20, 2020, a partial lockdown was announced in Bavaria, Germany, due to the corona pandemic, leading to a rapid shutdown of the outpatient unit of the Psychiatric District Hospital Regensburg (13). Within 10 days, the online blog “Bleib Zuhause” (German for “Stay at home”) (www.medbo.de/bleibzuhause) was launched with its first article being published on March 30 2020. On the first days, informative articles were published containing information about reliable sources of information concerning Covid-19, about rules of conduct for everyday life in order to stop the rapid spread of the virus, about face masks and current procedures within the hospital. On April 3, 2020, the regular blog activity started, presenting two articles per day. After 10 days, the 1-h-limit of article availability was changed, as it turned out to be too short for the blog's readers. From then on, all articles were visible for the whole day of their publication. From May 25, 2020 to June 1, 2020, the feedback survey was online. After relaxation of the lockdown measures, blog activity was reduced to one article per working day, from June 2, 2020 onwards. On July 1, 2020, the blog was stopped for a summer break.

### Announcement of the Blog

In order to inform patients about the launching of the blog, it was mentioned in scheduled phone calls. In addition, flyers and posters were printed and made available. Flyers were also added to regular mail to patients (e.g., prescriptions). Additionally, local newspapers and local TV and radio stations reported on the blog in order to bring the news to its potential recipients.

### Page Views

**Figure 1:**
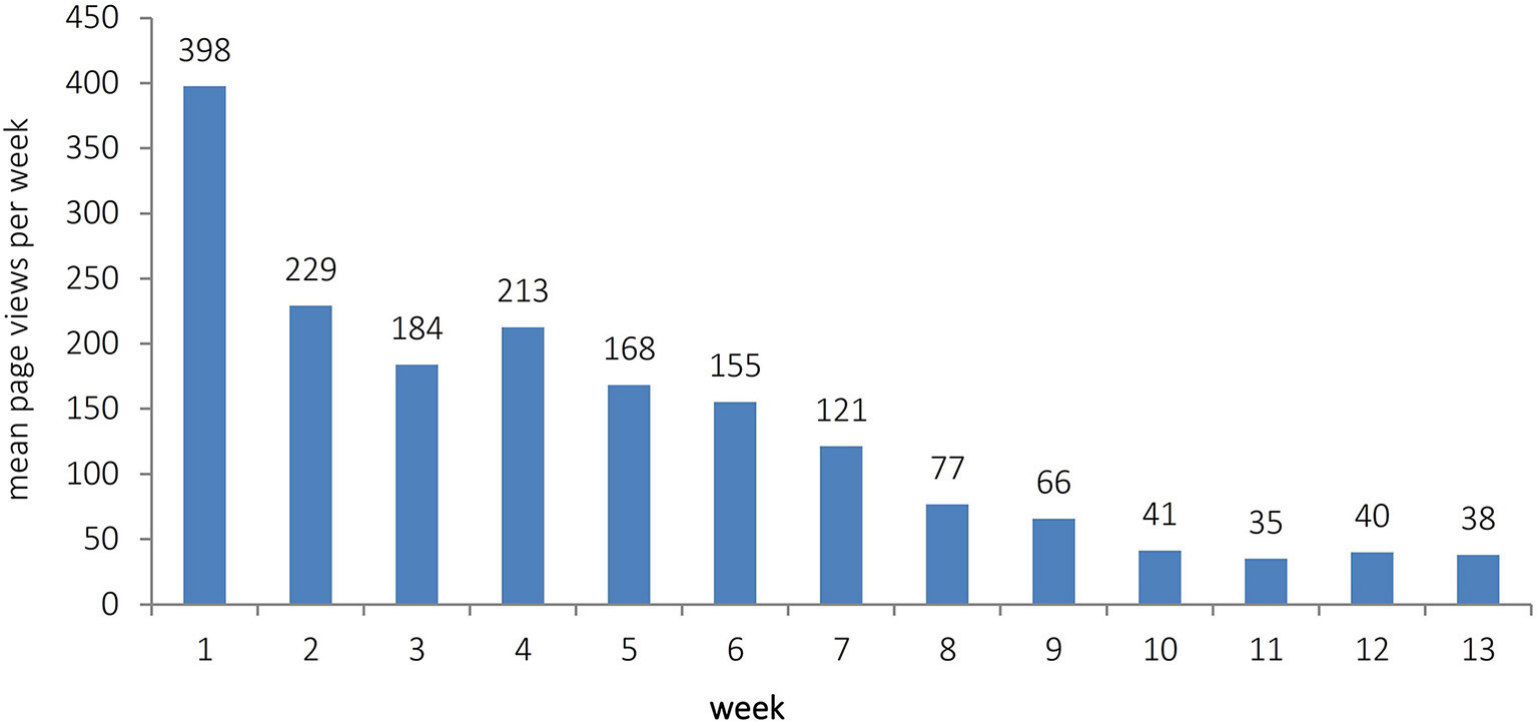
Mean page views for all 13 weeks, in which the blog was online.

Overall, the blog was viewed 12.358 times from April 1, 2020 to June 30, 2020. There were a mean of 135.80 page views per day. The blog was mostly viewed from Monday to Friday with least views on Sunday (see Table 1). The unpaired *t*-test comparing page views on weekdays (155.06 ± 127.20) with page views on weekends (87.65 ± 85.10) showed a statistically significant difference [*t*(89) = 2.485; *p* = 0.015]. In Figure 1, mean page views for all 13 weeks during which the blog was online, are depicted.

**Table 1:** Mean page views per weekday.

	**Mean page views ± standard deviation**
Monday	166.31 ± 147.37
Tuesday	166.85 ± 148.34
Wednesday	150.69 ± 95.36
Thursday	140.62 ± 102.27
Friday	150.85 ± 149.89
Saturday	100.77 ± 95.56
Sunday	74.54 ± 74.72

### Results of the Online Survey and Patient Feedback

Ten readers answered the survey, nine of them being female. All reported to have experienced psychiatric illness before. All of them had been under medical treatment in the Psychiatric District Hospital Regensburg and also lived within the area served by the hospital. The change of clinical subjective global impression can be found in Table 2.

**Table 2:** Change of clinical subjective global impression in comparison to pre-lockdown (simple frequency count; *n* = 10).

	**Somatic wellbeing**	**Mental wellbeing**
Very much better	0	1
Much better	0	0
Better	2	1
No change	3	2
Worse	4	5
Much worse	1	1
Very much worse	0	0

Reasons for worsening were social isolation, weight gain, worsening of pain due to missing physiotherapy and the need to wear a face mask. Reasons for improvement were going for a walk more frequently (going for a walk individually was allowed in Bavaria at all times), unexpected support by others and stress reduction.

With respect to the blog, seven of ten readers knew and read the blog from the very beginning. Six readers said the blog was helpful or very helpful, three said it was “neutral,” one did not answer the question. The frequency of blog visits, reads and active usage of the content is summarized in Table 3. When asked about useful aspects of the blog, readers named relaxation techniques, variety of content as well as the knowledge that “someone is interested in me and my need for support.”

**Table 3:** Frequency of blog usage (self-report by the readers).

	**How often do you visit the blog?**	**How often do you read the current articles?**	**How often do you transfer the information and tips to your everyday life?**
Twice a day	2	0	0
Daily	4	2	0
Every other day	3	6	3
Twice a week	0	1	3
Weekly	0	0	1
Less than once a week	0	0	2
Missing	1	1	1

Outside of the survey, some patients told us in phone calls or emails that the blog was like an Advent calendar for them, triggering positive feelings like curiosity and pleasant anticipation about the content that might be online next. All in all, however, the option to write an email if there were questions, comments or feedback concerning the blog content was scarcely used.

## Discussion

In times of the covid-19 pandemic, there is increasing awareness of the possibilities and advantages telemedicine has to offer: it helps protecting hospitals, vulnerable patient groups and health care professionals alike by limiting direct interpersonal contact (17). Furthermore, telemedicine ensures treatment continuity for chronically ill patients even in times of public lockdown (18). While telemedicine is mostly used in terms of phone or video calls, there are many additional resources which might be useful especially in times of a pandemic. There are many mental health-related websites known to be used by psychiatric patients (19). Therefore, there have already been ideas about telehealth services which support patients in their use of such online resources (20). In addition, there are many online blogs on different psychiatric diagnoses or on mental health in general written by patients themselves, psychiatrists or e.g., the American Psychiatric Association (APA) (21). Those blogs mainly contain individual reports on mental illness and recovery or general content on psychoeducation and current research. A huge advantage of blogs is their broader audience (21): Many patients are able to read, use and benefit from the same blog post. As blog posts can be read anonymously, being at home without anybody watching, patients may also be more willing and able to deal with the provided content in more depth; or experiment with exercises they would feel embarrassed about in the presence of others. On the other hand, only few blogs contain scientifically sound information. Moreover, in most cases blogs are written by authors that patients do not know and with whom they have no direct personal relationship. Our approach set out to combine the possibility of reaching a broader audience, i.e., the clinic's patients as a whole, as well as providing content by authors that patients already knew from their usual treatment sessions and with whom there already existed a therapeutic relationship. Content could be used in addition to personal or tele-appointments by providing e.g., current information, psychoeducation, tutorials for exercises or ideas for daily healthcare routines.

The blog aimed at supporting patients suffering from chronic psychiatric disorders during public lockdown, by providing daily content from different therapeutic professions and thus helping to maintain a daily routine. It was used as an add-on to scheduled tele-appointments and was by no means meant to replace one-to-one-contact or regular therapies. Due to the rapid spread of coronavirus and the sudden need of support for mentally ill patients who were instructed to stay at home, a quick start of the blog was given top priority.

There were both advantages and disadvantages implicated by the pressure of time that have to be kept in mind. The blog's content was created by the clinic's healthcare staff. An important advantage of this approach was that information was given by therapists that patients were already acquainted with from their regular treatment sessions, which might have fostered the feeling of still being connected despite the need to stay at home. Furthermore, it was hoped that ideas and tips from familiar healthcare staff might be taken seriously and therefore result in more active behavior than general tips found on the internet. However, these assumptions were not evaluated and have to be seen as hypotheses requiring future investigation. A disadvantage of having clinical staff create blog content was that obviously this was not their usual field of activity. Therefore, audios and videos produced could not meet professional standards but were of amateur quality. Furthermore, some efforts had to be made to win staff for the project within a few days. Some of them reacted with initial uncertainty concerning the creation of all-available online content that could be read by anyone interested. Nevertheless, many therapists were highly motivated to contribute to the blog since they felt a strong need to support their patients during the lockdown period. In order to address the existing uncertainties, it was especially important for the staff to (a) be thoroughly informed about the blog and about how the content will be used, (b) get technical support if needed for audio or video recordings, and (c) have free choice concerning the medium they desired to use: Some therapists decided against self-made videos in favor of text-based content including links to online-resources. After some blog articles had been published, the willingness to add content increased markedly, resulting in about 15–20 therapists from different disciplines contributing single or multiple articles to the blog.

A huge challenge was limited contact possibilities to patients during lockdown which made it hard to inform them about the launch of the blog. Of course, the blog was mentioned in scheduled phone calls. However, as many regular therapies had already stopped and phone calls could only cover a small amount of those therapies, many patients were not regularly contacted by the clinic within the first days of lockdown. Therefore, additional measures as stated above (flyers etc.) had to be taken to inform patients about the initiative. Additionally, it became clear that, although usually participating in the same therapy groups, patients were barely staying in contact with each other outside of treatment settings. Consequently, it was hardly spread by word of mouth or social media contacts between patients that the clinic offered the blog as support for everyone in need. Presumably the still existing stigma of psychiatric disorders prevents patients to share content in social media related to their role as a patient of a psychiatric clinic.

All in all, we realized that psychiatric patients are not well connected among each other by social media and it would have been much easier to reach patients if there had been a mailing-list with the permission to use it for such purpose.

With respect to page views, there were more views on weekdays than on weekends, which might mainly have been caused by the schedule of the blog (only one blog post on Saturdays and no new post on Sundays). On Sundays, only the archive containing informative blog posts was available. Consequently, an archive does not appear sufficient to reach patients on a daily basis in order to support them in their everyday routine. In this regard, very regular posts seem to be necessary.

The development of views in the course of time shows an initial peak of page views. In the first week, many efforts were made to inform patients about the blog's existence. As patients could not be contacted directly due to data protection issues, the news about the blog was spread using local radio and newspaper. Therefore, in the first week, many readers might have viewed the blog that were not necessarily part of its primary target group, i.e., individuals affected by chronic psychiatric illness. After about 4 weeks, a downward trend of page views started. This coincided with the time when in Bavaria, the partial lockdown was relaxed a bit and people were again allowed to leave their houses without cause. This and maybe a certain tiredness with respect to coronavirus-associated issues or a habituation to the situation may have gradually reduced the interest in online content.

We also experienced that patient feedback or ideas concerning the blog were hard to come by. Only 10 readers filled in the survey and only about 25 emails in total were written containing feedback, questions or suggestions concerning blog content. This may, at least in part, be due to characteristics of the patient group addressed: patients suffering from severe and/or chronic psychiatric conditions who might have little belief in their self-efficacy and who might not have been confident that their feedback was of real interest, or was able to change the blog's content. If asked during scheduled appointments, many patients gave very positive feedback with respect to the blog. We therefore assume that giving feedback online or via email was difficult for many patients – maybe as they did not know who exactly was receiving the message or maybe because of a fear to reveal sensible information by writing an email (i.e., coming out as someone who reads a blog for patients with psychiatric disorders). After all, the blog was conceptualized as an interactive project including both input from the clinic on the one side, and patient involvement on the other. We are still convinced that in phases of public restrictions and lockdown, offering a blog or some other form of interactive, low-threshold online-program including a lively exchange with and active involvement of patients would be desirable. However, as is already known from other forms of content on the internet, active reader involvement is not easy to accomplish and there is an urgent need for more sophisticated structures and tools to make patient involvement easier and more attractive. It may be of particular importance to offer tools which enable an anonymous, yet interactive, form of interaction. Thus, even if one would assume that the possibilities offered by the various forms of electronic communication might facilitate interaction among patients, patient empowerment and patient involvement, one has to realize that this might be more difficult to achieve for psychiatric disorders as compared to other chronic diseases. Therefore, the establishment of efficient communication structures for patient involvement in psychiatry presents a challenge that needs to be addressed in the future.

The little feedback on the blog considerably limits the conclusions that can be drawn from the added benefit the blog might have had on the regular treatment of patients during periods of lockdown. Nevertheless, the numbers of blog visits indicate that a considerable number of patients was reached during the time of restrictive anti-coronavirus measures. The authors are well aware about the limitations of this paper and the preliminary nature of the project. The blog was set up to address a suddenly arising need and with the aim to offer support for our patients as quickly as possible. We decided to aim for a publication of our experience with this project, hoping that everyone interested in supporting psychiatric patients in times of lockdown public health measures might benefit from our ideas and practical insights.

In sum, it was possible to launch a blog within few days, being supported by clinical staff, leading to positive patient feedback patients. Based on the daily visits, it can be assumed that the blog had a constant readership. Furthermore, different patients repeatedly reported that the feeling of the clinic still being interested in their wellbeing was an important and helpful message of the blog during the shutdown of much of public and private live. However, given the relatively limited feedback received, including a low response rate to the final online survey, we have to assume that only a small part of ideas and tips given in the blog were really translated into active behavior. Moreover, the blog didn't reach as many patients as was hoped, and overall, patient involvement turned out to be the most challenging part of the project. The blog did not generate its own considerable, interactive momentum, but lived from the clinic's input. Further development of this format including more sophisticated structures and tools will be necessary to make involvement by the target group more attractive.

As the blog was launched and operated by the clinic's health care staff who returned to full-time work after the end of the most restrictive lockdown measures, there were no resources anymore available to continue with the blog intensively. Therefore, it was not possible for the clinic to further improve and develop the format. In all, it's our hope that this paper will raise awareness for the situation of psychiatric patients during the Covid-19-pandemic and for the need for the development and improvement of low-threshold tele-health interventions for this particular patient group.

## Data Availability Statement

The raw data supporting the conclusions of this article will be made available by the authors, without undue reservation.

## Ethics Statement

The studies involving human participants were reviewed and approved by Universität Regensburg, Ethikkommission, D-93040 Regensburg. Written informed consent for participation was not required for this study in accordance with the national legislation and the institutional requirements.

## Author Contributions

AL managed and coordinated the online blog, collected and analyzed data, wrote the manuscript. BL conceived of the present idea, supervised the project and contributed ideas to the online blog. KN introduced patients' perspective to the project. WS helped with project coordination and supervision of the project. All authors contributed ideas to the online blog, discussed the results and contributed to the final manuscript.

## Conflict of Interest

The authors declare that the research was conducted in the absence of any commercial or financial relationships that could be construed as a potential conflict of interest.
